# Rare Encounter With Hepatic Epithelioid Hemangioendothelioma: A Case Report

**DOI:** 10.7759/cureus.80567

**Published:** 2025-03-14

**Authors:** Mariam Malik, Rana Bilal Idrees, Zeeshan Mirza, Sadia Anwar, Barira Ahmad, Muhammad Hamid Chaudhary

**Affiliations:** 1 Department of Radiology, Nuclear Medicine, Oncology and Radiotherapy Institute-Atomic Energy Cancer Hospital (NORI-AECH), Islamabad, PAK; 2 Department of Radiology, Institute of Nuclear Medicine and Oncology-Atomic Energy Cancer Hospital (INMOL-AECH), Lahore, PAK; 3 Department of Cardiac Surgery, Chaudhry Pervaiz Elahi Institute of Cardiology, Multan, PAK

**Keywords:** case reports, diagnostic imaging, gastrointestinal neoplasms, hepatic epithelioid hemangioendothelioma, liver neoplasms

## Abstract

Malignant hepatic epithelioid hemangioendothelioma (HEHE) is a rare vascular tumor with variable malignant potential, often affecting middle-aged individuals. Hepatic involvement is uncommon, presenting diagnostic challenges due to overlapping imaging features with other liver pathologies. We report a case of a 35-year-old female with HEHE initially presenting with epigastric pain and an incidental liver mass. Imaging revealed multifocal hepatic lesions, confirmed as HEHE through biopsy and immunohistochemistry. Despite initial stability on tamoxifen, disease progression occurred after seven years, manifesting as an increase in the size and number of hepatic lesions. Hepatic transplantation was planned, which was refused by the patient. Subsequently, the disease progressed to a large confluent hepatic mass with calcifications, lymphadenopathy, and pulmonary metastases. Systemic chemotherapy was henceforth initiated. This case underscores the importance of a multimodal approach integrating imaging, histopathology, and tailored therapeutic strategies for diagnosing and managing HEHE. Early detection and comprehensive management are critical to improving outcomes where liver transplantation offers a potential cure.

## Introduction

Malignant epitheliod hemangioendothelioma is a tumor of variable malignant potential that arises from vascular endothelium [1] with biologic characteristics ranging between benign hemangioma and malignant angiosarcoma [2]. The pathology is exceptionally rare [3] and accounts for 1% of all primary liver tumors. It has an unpredictable course and a propensity for aggressive behavior. Due to its rarity, limited cases have been reported in the literature, making its diagnosis and management challenging. This tumor may arise in the liver, lungs, breast, bones, head and neck regions, lymph nodes, and skin [4]. The common age of presentation is between 30 and 50 years [4]. 

Involvement of the liver is infrequent [2] with a reported frequency of 0.1 in 100,000 of the population worldwide [5] and a female-to-male ratio of 3:21. The right lobe of the liver is more commonly involved compared to the left lobe [6]. Some of the risk factors associated with the development of hepatic epitheliod hemangioendothelioma (HEHE) include alcohol intake, contraceptive pills, vinyl chloride, and hepatic trauma [7]. The clinical presentation can range from being asymptomatic to right upper quadrant pain [7], hepatic malfunction [1], and even Budd-Chiari syndrome. 

Imaging plays a crucial role in diagnosis, tumor staging, assessment of response to therapy, identification of disease resolution after completion of treatment, and tumor recurrence. The modalities used for evaluation include ultrasound, triphasic liver CT/MRI, and PET-CT; however, establishing an absolute diagnosis requires histopathological review with immunohistochemistry. Some of the factors associated with adverse prognosis include male gender, age of more than 55 years, increase in the size of disease, the presence of ascites, pulmonary involvement, and multiorgan involvement [8].

Early disease is seen as discrete nodules on imaging, whereas in its more advanced stages, the lesions may confluence to form diffuse masses invading the hepatic vasculature and reaching up to the liver capsule. There may be fibrosis with compensatory hypertrophy of the uninvolved liver parenchyma and consequent capsular retraction. Because the tumor is capable of metastasis and has an increased probability of recurrence, imaging is essential in treatment planning and follow-up. 

We now present a case from our department of a non-resectable HEHE patient who exhibited progressive disease despite treatment. Given the extent of the disease and its resistance to conventional therapies, liver transplantation was recommended as the next step in management. However, the patient declined surgical treatment and continued with medical therapy. Despite being compliant, she subsequently developed metastatic disease.

## Case presentation

**Figure 1:**
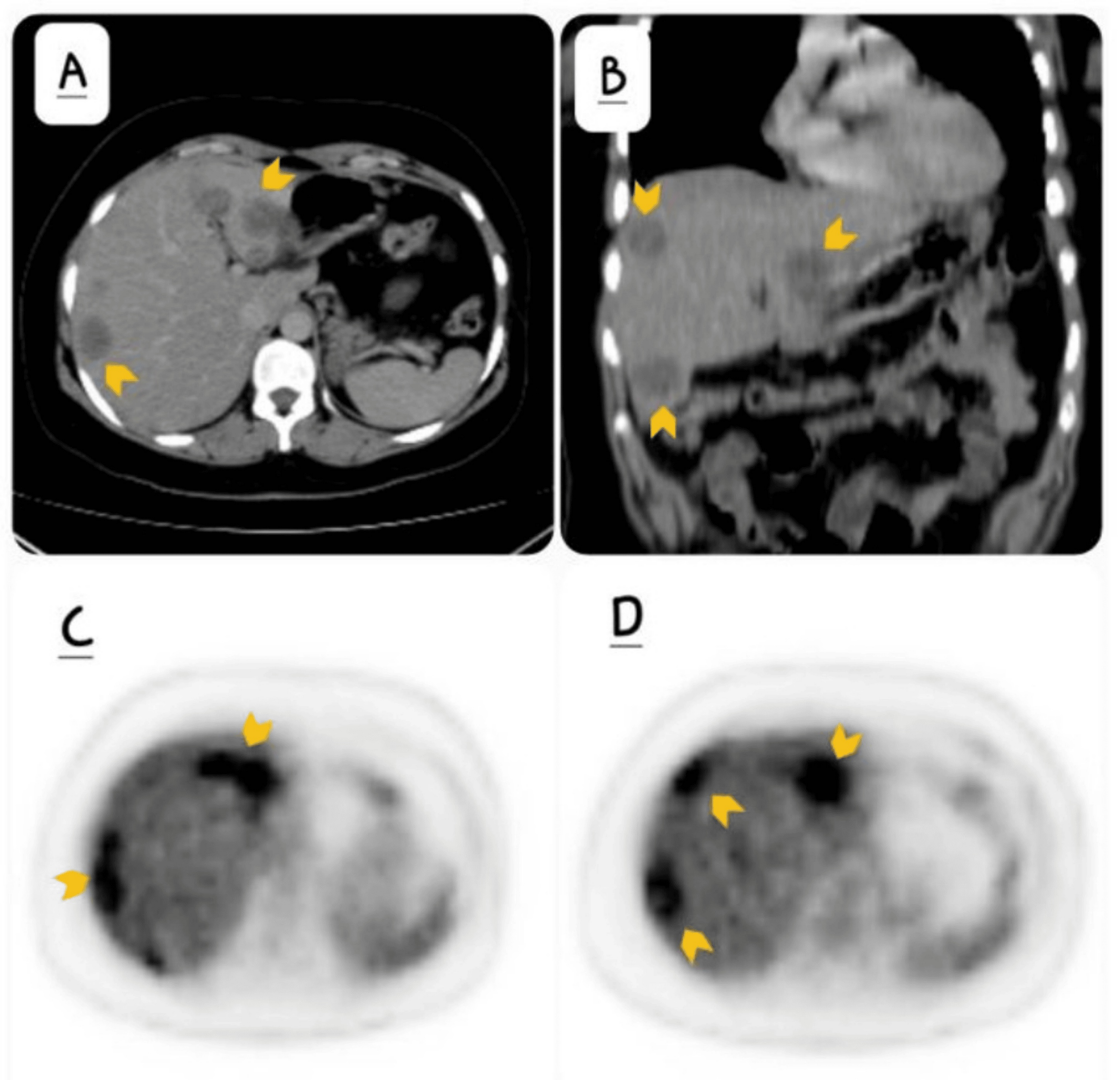
Imaging findings A: axial post-contrast computed tomography (CT) of the abdomen and B: coronal post-contrast computed tomography (CT) demonstrating well-circumscribed hypodense lesions in both lobes of the liver (yellow arrows); C, D: axial fluorodeoxyglucose-positron emission tomography (FDG-PET) confirmed these lesions to be hypermetabolic (yellow arrows).

A 35-year-old female who underwent cholecystectomy for cholelithiasis in 2016 presented after a few months with epigastric pain, nausea, and vomiting. Ultrasound (USG) of the abdomen as an initial investigation at a local facility revealed an incidental finding of a liver mass. She was then referred to the oncology outpatient department, where further workup was advised. Computed tomography (CT) showed multiple hypodense lesions in both lobes of the liver (Figures 1A, 1B). Fluorodeoxyglucose-positron emission tomography (FDG-PET) confirmed these lesions to be hypermetabolic (Figures 1C, 1D). No distant metastasis was identified.

**Figure 2:**
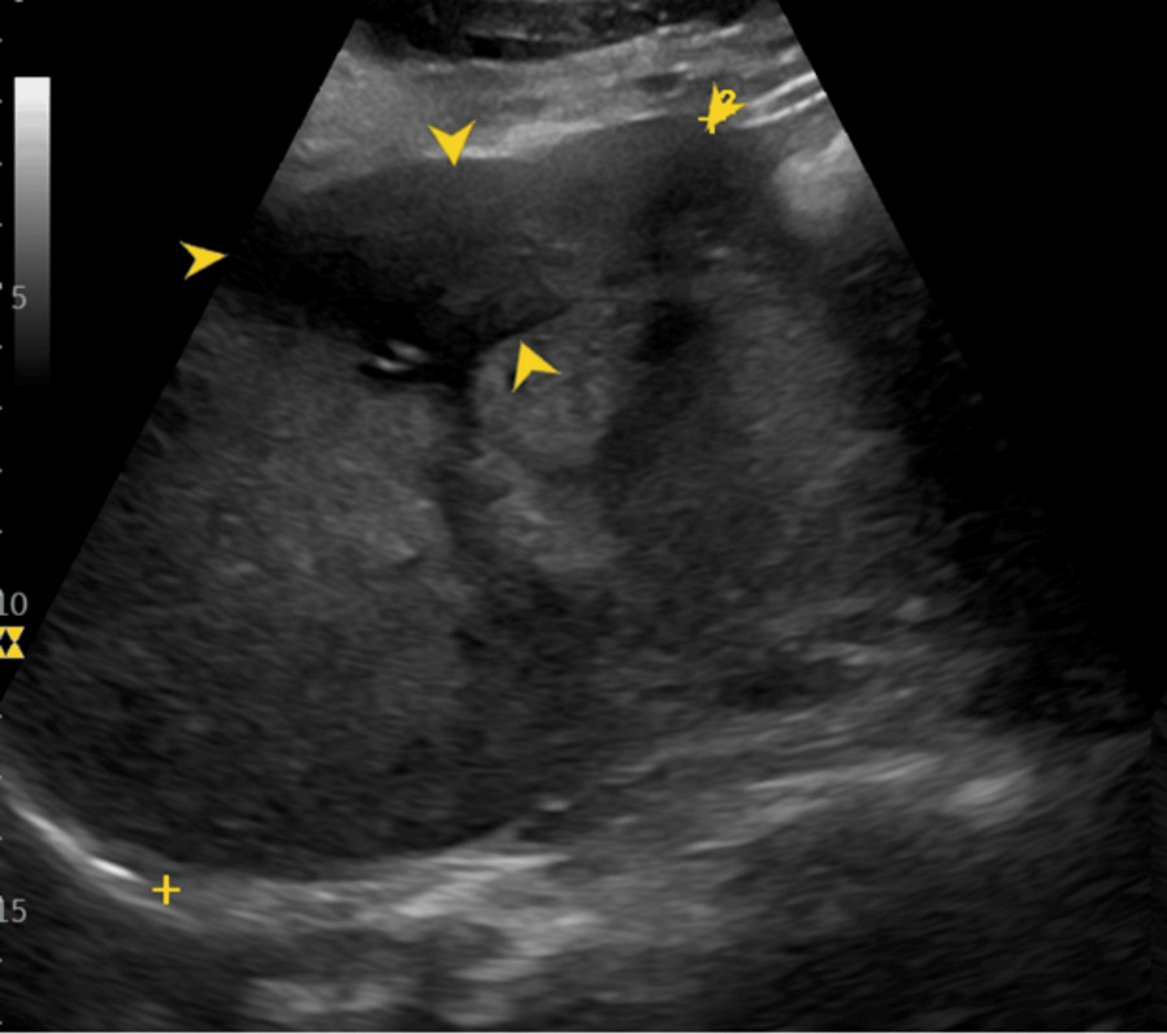
Gray-scale ultrasound image This demonstrates a large, hypoechoic liver lesion at a subscapular peripheral location annotated by the yellow arrows.

Subsequent USG-guided core biopsy showed a tumor composed of epithelioid cells with cytoplasmic vacuolation, hypochromasia, and pleomorphism, confirming the diagnosis of HEHE. Immunohistochemical staining of tumor cells was positive for cluster of differentiation-34 (CD34), cluster of differentiation-31 (CD31), and estrogen receptors. The patient was started on tamoxifen and advised serial ultrasounds every six months. The patient remained well for seven years, after which she developed an increase in the number and size of hepatic parenchymal lesions confirmed on ultrasound, despite being compliant with medical therapy. She was advised to have a liver transplantation at this time; however, she refused surgery and got lost to follow-up, only to present six months later with increasing abdominal distention. On examination, the liver was enlarged, and abdominal USG (Figure 2) showed an irregular, ill-defined, heterogeneous echogenicity mass with internal echogenic foci indicative of intralesional calcification.

**Figure 3:**
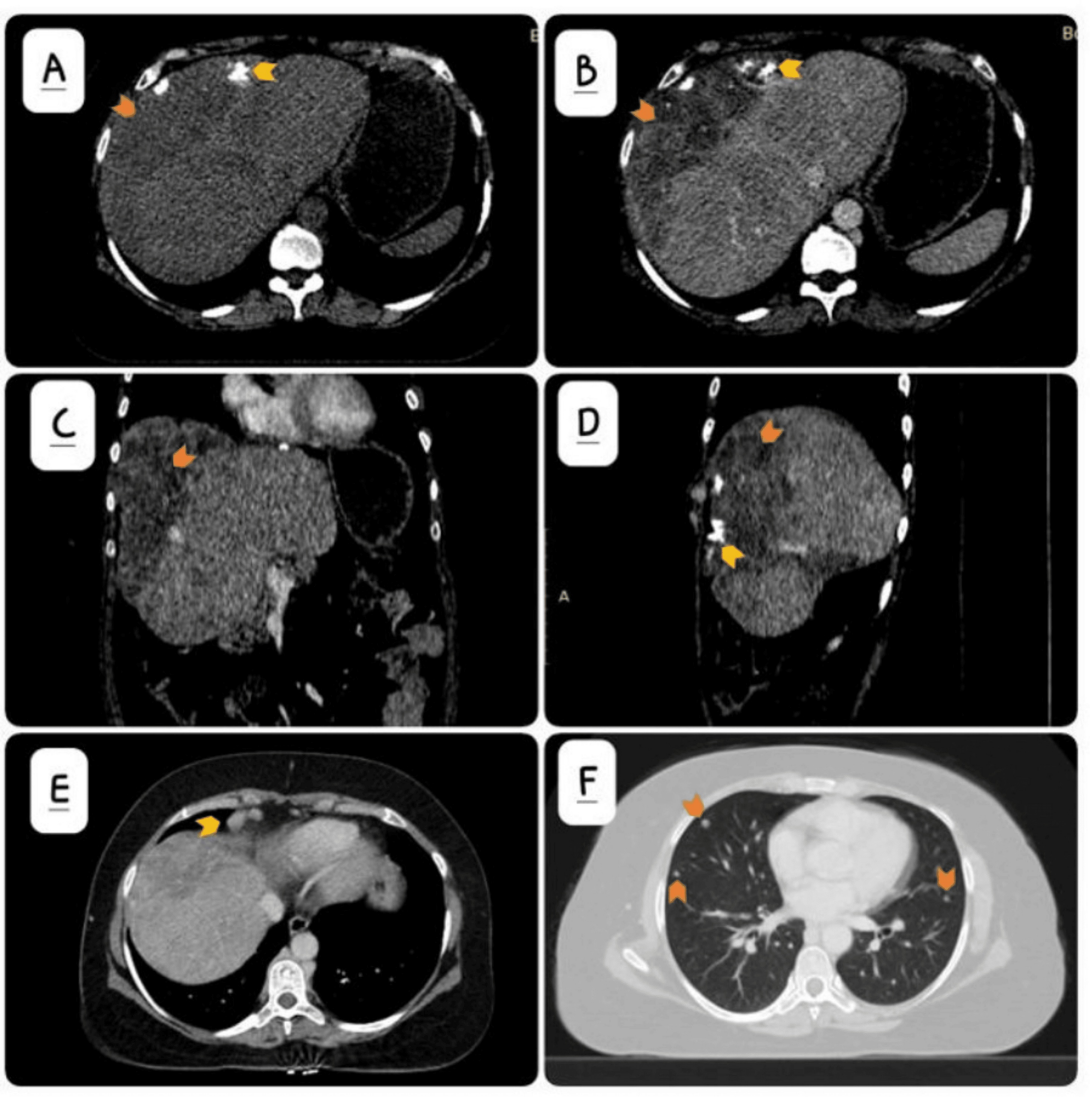
Triphasic contrast-enhanced computed tomography findings A: non-contrast axial CT image demonstrates a large subcapsular confluent hypodense lesion (orange arrow) with chunky calcification (yellow arrow); B: contrast-enhanced axial; C: coronal; and D: sagittal CT images in the portovenous phase show patchy enhancement (orange arrow) and intralesional calcification (yellow arrow); E: contrast-enhanced axial CT image shows enlarged lymph nodes at the right superior diaphragmatic location (yellow arrow); F: soft tissue intrapulmonary nodules (orange arrows) concerning for metastasis.

Triphasic contrast-enhanced computed tomography with a liver protocol showed a large, confluent hypodense lesion involving both lobes of the liver, predominantly in a subcapsular peripheral location with chunky calcifications (Figure 3A-3D). This lesion, which was formed by confluencing previous smaller lesions, showed marginal enhancement on the arterial phase, with progressive filling on the portal venous and delayed phases. Additional smaller well-circumscribed lesions were also present in both lobes. There was an associated development of enlarged right superior diaphragmatic lymph nodes (Figure 3E). There was the development of multiple randomly scattered intrapulmonary nodules suspicious for pulmonary metastasis (Figure 3F). An MRI abdomen with contrast could not be performed as the patient had an MRI-incompatible metal prosthesis in her right femur.

**Figure 4:**
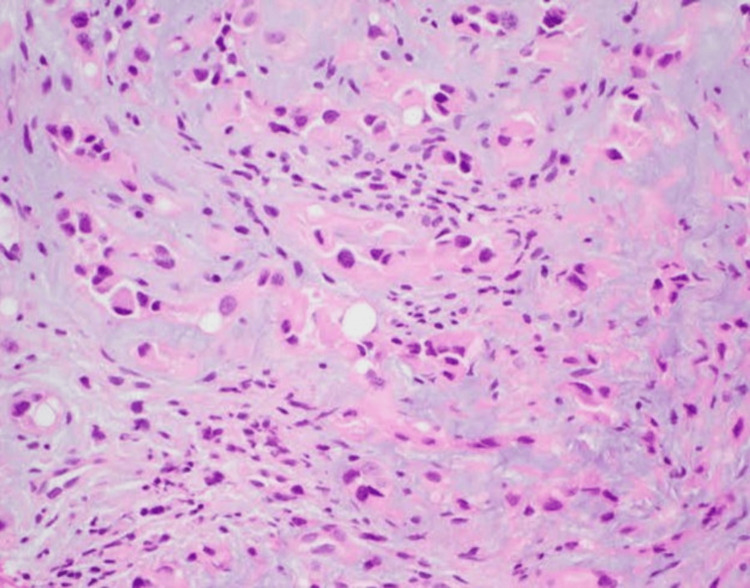
Hematoxylin and eosin (H&E) stain (20x magnification) from the liver lesion reveals cord, strands, and small nests of large endothelial cells with abundant eosinophilic cytoplasm embedded in a myxohyaline stroma. Tumor cells have vesicular, round-to-oval, and in some places indented nuclei.

Ultrasound-guided core biopsy was performed of the hepatic parenchymal lesion to reconfirm the diagnosis, which again showed it to be an HEHE (Figure 4). In addition, CT-guided core biopsy of the right superior diaphragmatic lymph nodes was positive for metastasis.

The pulmonary nodules were too small to be biopsied; however, since these were a new finding in comparison with previous imaging of 2016, they were also considered to be suspicious for metastasis. On follow-up CT after three months, these pulmonary nodules showed an increase in size and number, further confirming them to be of metastatic origin. The patient was referred to the oncology department, where she was started on systemic chemotherapy with doxorubicin and vincristine.

## Discussion

When HEHE is encountered, it is more often observed in middle-aged women with non-specific symptoms [1]. The pathology can be challenging to diagnose based on radiological characteristics alone, as imaging features may overlap with other liver lesions such as metastasis [1]. Ultrasound is usually the first modality advised for abdominal pain, which is the most common presenting complaint of patients with this condition. On ultrasound, these may be observed as multifocal distinct heterogeneous nodules of variable echogenicity. Intralesional calcification may be present, and color Doppler may demonstrate neovascularity. Capsular retraction can also be identified. 

Computed tomography (CT) usually demonstrates discrete or confluent peripheral/subcapsular lesions that are hypodense compared to background normal liver parenchyma. Non-contrast study aids in visualization of bulk of tumor, associated capsular retraction, and intralesional coarse calcification. Capsular retraction, however, is a non-specific finding, as this is also seen in other pathologies such as cholangiocarcinoma and metastasis. On post-contrast CT scans, there are three different patterns of enhancement seen with HEHE. Tumors of less than 2 cm in size exhibit homogeneous enhancement on the arterial phase, which persists on portal venous and delayed imaging; lesions between 2 and 3 cm in size reveal marginal enhancement on the arterial phase with complete filling in portal venous and delayed phases, which is also known as the “halo sign,” and larger lesions of size 3 cm or more reveal heterogeneous enhancement that progresses on portal venous and delayed phases. The infiltration of veins, venules, and sinusoids results in their obstruction with a resultant narrowing of the hepatic and portal vein branches as they advance towards the lesion. The occluded vessel gives the resemblance of a stick of lollipop, while the hypodense tumor appears like candy, a sign known as the “lollipop sign,” which is very specific for HEHE. Ahmad Alomari first described this in a 2006 case report in the European Journal of Radiology [9]. 

Magnetic resonance imaging (MRI) is superior to CT in detecting small lesions, especially if they are subcapsular. MRI demonstrates a “halo sign” in HEHE lesions with a central hypointense and marginal hyperintense signal on T1-weighted images and a central hyperintense marginal hypointense rim on T2-weighted sequences. The central hypointense signal on T1 may be due to sclerosis, hemorrhage, or calcification. The demarcation between normal hepatic parenchyma and HEHE may be difficult on all sequences; however, post-contrast T1 with gadolinium-based contrast media sharply interprets the extent of the tumor. Again, the three patterns of enhancement seen in contrast-enhanced CT may be appreciable on contrast-enhanced MRI depending upon the size of the tumor. Diffusion-weighted imaging (DWI) at high b-values demonstrates a high signal at tumoral margins and lower signals in the tumor core [10,11]. Apparent diffusion coefficient (ADC) reveals a hyperintense tumor core and hypointense tumor margins. The role of MRI in distinguishing HEHE from cholangiocarcinoma and metastasis is limited due to overlapping characteristics. However, HEHE demonstrates higher ADC values compared to hepatocellular carcinoma (HCC) (but lower than the normal liver parenchyma) as it has intermediate cellularity and abundant myxoid stroma compared to HCC, which is highly cellular. Even then, DWI alone is not definitive, and contrast-enhanced MRI features with histopathology remain integral for establishing the diagnosis.

There is variable fluorodeoxyglucose (FDG) uptake on positron emission tomography (PET) scan, which usually is moderate [12]. When metastasis is present, these may be observed in abdominal lymph nodes, omentum, mesentery, lungs, muscles, and skin. Mortality is higher in patients with metastasis.

Histopathology is the gold standard for diagnosis, and in our patient, a pre-treatment diagnosis was made by ultrasound-guided core biopsy of the largest lesion in the right lobe assisted with immunohistochemistry. Possible differential diagnoses of HEHE may include cholangiocarcinoma, metastatic hepatocellular carcinoma (HCC), or angiosarcoma [13]. Findings in tissue diagnosis that favor HEHE include intermediate, dendritic, or epithelioid cells where dendritic cells demonstrate a stellate appearance, epithelioid cells are seen in all lesions of endothelial origin, and intermediate cells show attributes of both cell types [6]. Microvascular channels containing red blood cells may also be seen. Epithelioid cells demonstrate a cluster of differentiation-31 (CD31), cluster of differentiation-34 (CD34), and calmodulin-binding transcription activator 1 (CAMTA 1) in immunohistochemistry [14]. Differentiation from angiosarcoma may be difficult; however, features like higher mitotic activity, nuclear pleomorphism, and greater atypia favor angiosarcoma, while a lesser degree of hepatic parenchymal involvement and greater sclerosis favor HEHE6. Cholangiocarcinoma, metastasis, and HCC stain negative for CD31, CD34, and factor VIII, and HCC stains positive for cluster of differentiation-10 (CD10), arginase, and hepatocyte paraffin 1 (HepPar-1) with polyclonal carcinoembryonic antigen [6]. 

Many treatment options are available for HEHE, which include chemotherapy, immunotherapy, transcatheter arterial embolization (TAE), ablation, surgical resection, or liver transplantation. Surgical excision is the primary curative treatment option in the absence of metastasis; however, in cases of multifocal or large lesions involving multiple liver segments or both lobes, liver transplantation is the required available curative treatment option. The prognosis depends on tumor grade and is better for slow-growing lesions. Liver transplantation is associated with a five-year survival rate of up to 80% [15]. No proper follow-up protocol is available because of the rare incidence of this condition.

## Conclusions

Hepatic epithelioid hemangioendothelioma (HEHE) remains a rare and diagnostically challenging vascular tumor with a clinical spectrum ranging from indolent presentations to aggressive, multifocal disease. This case report underscores the critical role of multimodality imaging in identifying characteristic features, such as the "halo sign" and "lollipop sign," while emphasizing that definitive diagnosis relies on histopathological confirmation with immunohistochemical markers. Despite initial conservative management, the progression to metastatic disease in this patient highlights the limitations of current treatment strategies. Ultimately, a multidisciplinary approach, including consideration for liver transplantation in non-resectable cases, is essential to optimize patient outcomes in the face of this rare malignancy.
